# Stroke, Seizures, Hallucinations and Postoperative Delirium as Neurological Complications after Cardiac Surgery and Percutaneous Valve Replacement

**DOI:** 10.3390/jcdd9110365

**Published:** 2022-10-24

**Authors:** Johannes Teller, Maria Magdalena Gabriel, Svea-Dorothee Schimmelpfennig, Hans Laser, Ralf Lichtinghagen, Andreas Schäfer, Christine Fegbeutel, Karin Weissenborn, Carolin Jung, Lukas Hinken, Hans Worthmann

**Affiliations:** 1 Department of Neurology, Hannover Medical School, 30625 Hannover, Germany; 2 Department of Anaesthesiology and Intensive Care Medicine, Hannover Medical School, 30625 Hannover, Germany; 3 Department for Educational and Scientific IT Systems, Centre for Information Management, Hannover Medical School, 30625 Hannover, Germany; 4 Institute of Clinical Chemistry, Hannover Medical School, 30625 Hannover, Germany; 5 Cardiac Arrest Center, Department of Cardiology and Angiology, Hannover Medical School, 30625 Hannover, Germany; 6 Department of Cardiothoracic, Transplantation and Vascular Surgery, Hannover Medical School, 30625 Hannover, Germany; 7 Volkswagen AG, 38440 Wolfsburg, Germany

**Keywords:** cardiac surgery, percutaneous valve replacement, seizure, hallucination, stroke, postoperative delirium

## Abstract

(1) Background: Neurological complications such as acute ischemic stroke or postoperative delirium are frequent after cardiac surgery or percutaneous valve replacement. This study aimed to identify corresponding risk factors. (2) Methods: 297 patients with percutaneous valve replacement or cardiac surgery were postoperatively screened for neurological complications such as delirium, stroke, seizures and hallucinations twice daily for three days. Pre- and perioperative risk factors were evaluated in a multivariate model. (3) Results: Neurological complications occurred in 43.8% (*n* = 130) as composed of delirium (43.43%, *n* = 129), stroke (2.7%, *n* = 8), seizures (1.35%, *n* = 4) and real hallucinations (3.36%, *n* = 10). Multiple logistic regression revealed an association of neurological complications with lower Montreal Cognitive Assessment scores (Exp(B) 2.042; 95% CI, 1.183–3.525, *p* = 0.010), older age (Exp(B) 1.071; 95% CI, 1.036–1.107, *p* < 0.001), red blood cell transfusions until postoperative day 3 (Exp(B) 1.157; 95% CI, 1.030–1.300, *p* = 0.014), history of heart failure (Exp(B) 1.985; 95% CI, 1.130–3.487, *p* = 0.017) and increased CRP levels (Exp(B) 1.004; 95% CI, 1.000–1.008, *p* = 0.037). (4) Conclusions: Postoperative delirium remains a frequent complication after cardiac surgery, while stroke and seizures occur rarely. A preoperative risk profile including older age, history of heart failure and cognitive impairment was identified for a complicated postoperative course. However, the impact of an intense inflammatory response must not be neglected.

## 1. Introduction

Cardiac surgery and percutaneous valve replacement are severe interventions with a high risk of peri- and postoperative complications [1]. Neurological complications namely acute ischemic stroke (AIS), focal or generalized seizures and hallucinations after surgery are known to be associated with poor outcome [2,3]. Previous studies showed that 1.5 to 4.5% of patients suffer from AIS after cardiac surgery [4,5]. Vedel et al. reported clinically silent AIS in more than 52% of 197 enrolled patients after cardiac surgery as detected by diffusion-weighted MRI [6]. In addition to cerebrovascular complications, up to 52% of the patients after cardiac surgery develop an episode of postoperative delirium (POD) [7]. Patients with these neurological complications showed longer intensive care unit (ICU) and hospital stays as well as higher in-hospital mortality compared to those without [8]. Furthermore, POD is associated with high costs, poor functional recovery, functional decline and low postoperative cognitive function [9]. Neurological complications remain often unrecognized after surgery. This is especially the case for the hypoactive type of delirium [10]. Prospectively collected data about hallucinations, AIS or seizures after cardiac surgery or percutaneous valve replacement are scarce [2,6]. In particular, data comparing cardiac surgery and percutaneous valve replacement as well as structured data about pseudo-hallucinations are missing. The aim of the study is to prospectively describe the various neurological complications after cardiac surgery or percutaneous valve replacement and to identify possible risk factors. Hence, this study could reveal a rationale for the importance of systematic screening for neurological complications after surgery and lead to a better recognition of risk factors.

## 2. Materials and Methods

### 2.1. Study Population

In a prospective monocentric study, adult patients who underwent elective cardiac surgery (bypass, aortic and/or valve surgery) or elective percutaneous valve replacement (transcatheter aortic valve implantation or catheter based mitral valve reconstruction (MitraClip^®^, Abbott Vascular, Chicago, IL, USA) were preoperatively recruited at Hannover Medical School between August 2018 and March 2019. The study was approved by the local ethics commission (Ethics Commission of Hannover Medical School, Hannover, Germany, Approval No. 7876 BO S, Date of Approval 18 July 2018). Exclusion criteria were acute infection, emergency or revisional surgery, malignoma or chemotherapy in the last two years, dementia, known immunodeficiency and abusive alcohol or drug use as well as acute focal neurological deficits.

A detailed medical history of the patients with focus on neurological, psychiatric and cardiovascular pre-existing conditions was collected. Prior to surgery patients were examined neurologically to detect any preoperative deficit. Cognitive function was rated using the Montreal Cognitive Assessment (MoCA). Its’ result was categorized in the four official classifications: 1 = no (26–30 points), 2 = mild (18–25 points), 3 = moderate (10–17 points) and 4 = severe cognitive impairment (<10 points). For detection of the common risk factors of postoperative delirium depression and malnutrition the Patient Health Questionnaire-2 (PHQ-2) and the Malnutrition Screening Tool (MST), respectively, were used. Furthermore, history of preoperative-medication including self-medication, anaesthesiologic and intraoperative parameters were collected. Moreover, number of transfusions (RBC, fresh-frozen plasma, thrombocytes) until the third postoperative day (day 3), postoperative infections or other complications and postoperative medication were noted. Laboratory test results were taken from routine laboratory analysis. For the laboratory analysis the daily maximum and minimum values during the period from start of surgery to the third postoperative day were considered (day 0, day 1, day 2 and day 3). The pre-, intra- and postoperative data collection from the hospital information system was supported by the Enterprise Clinical Research Data Warehouse (ECRDW) maintained by the Centre of Information Management (ZIMt) of Hannover Medical School.

### 2.2. Screening of Neurological Complications

The neurological complications as composed of POD, AIS, focal or general seizures or hallucinations were assessed prospectively from the first postoperative day. POD was assessed using the Confusion Assessment Method (3D-CAM) or Confusion Assessment Method Intensive Care Unit (CAM-ICU) depending on the patients´ postoperative condition (Richmond Agitation-Sedation Scale (RASS)-Score −1 to +1 3D-CAM and otherwise CAM-ICU) [11]. The type of delirium was categorized by the RASS-Score in hypoactive (below 0), hyperactive (above 0) and mixed delirium (below and above 0). CAM-Severity long (CAM-S long, 0–19 points) was used to determine delirium severity for all patients with POD. Severe postoperative delirium was defined by the top quartile of CAM-S-long (≥11 points). In addition, subsyndromal delirium was evaluated, which is defined by two or more positive features of the four-feature delirium screening [12,13]. Also, the patients were interviewed for the occurrence of hallucinations, which were classified in real- or pseudo-hallucinations and visual or auditive type. Pseudo-hallucinations were diagnosed according to current definitions [14]: patients are able to distance themselves from the hallucinations and recognize that the hallucinations are not real. Pseudo-hallucinations can cause potentially compromising symptoms and may represent a precursor for real-hallucinations. AIS and focal or generalized seizures were detected by structured screening twice daily and additional examinations of the treating medical team in charge. If delirium was diagnosed or patients showed neurological symptoms, further diagnostics were initiated. The diagnosis of AIS or seizure was confirmed by clinical assessment, cerebral imaging and electroencephalography (EEG). In the case of AIS, symptoms were quantified by the National Institutes of Health Stroke Scale (NIHSS). The Trial of Org 10172 in Acute Stroke Treatment (TOAST) criteria were used for classification of the etiology of AIS. Patients were screened twice a day for three days (day 1 to day 3) postoperatively by a team of six qualified investigators. The first screening day was the first day after surgery. In case of prolonged unconsciousness, patients were screened instantly after regaining consciousness (*n* = 6). Apart from neurological screening, the postoperative level of pain was assessed using the numeric pain rating scale (NRS, 0–10 points). To achieve a 24 h coverage for the detection of neurological complications the nursing staff was instructed to pay special attention to neurological symptoms during the night.

### 2.3. Clinical Data Warehouse

For each individual subject coded discharge diagnoses-ICD-10-GM (International Classification of Diseases German Modification)-were extracted based on an entity recognition approach. Procedures applied within surgery were extracted and identified by the Operation and Procedure Classification System (OPS-301). Laboratory values with clinical relevance (*n* = 76) were selected by clinical experts. All data of the ECRDW were exported as a relational data basis and pivoted using Microsoft Power Query to be imported into SPSS.

### 2.4. Statistical Analysis

Statistical analyses were performed using SPSS for Windows (v26.0; IBM-Deutschland, Munich, Germany). Subjects were divided into two groups: patients with and without neurological complications. If not indicated otherwise categorical variables were reported in frequencies and continuous variables as mean ± standard deviation (SD) in case of normal distribution or as median with interquartile range (IQR) if data were non-normally distributed. In univariate analysis of baseline characteristics and follow up events, the Pearson Chi-square test was used for categorical variables. Group differences in continuous variables were evaluated using Mann-Whitney U-test and Student’s *t*-test for independent samples according to data distribution. Multivariate analysis adjustment was performed in case of evidence or suggestion for competing variables. Logistic regression by backward condition was used. The significance testing for all analyses was 2-sided with a type 1 error of 0.05 and a 0.95 confidence interval.

## 3. Results

**Figure 1:**
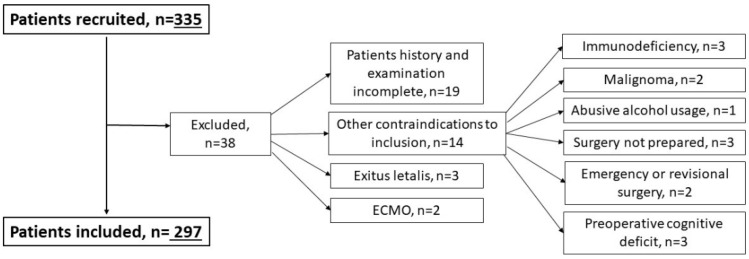
Section flow chart with reasons for study exclusion.

In total 335 patients were recruited of which 38 finally were excluded; 19 due to incomplete patient´s history or prospective screening, two due to deep sedation, three due to exitus letalis within the screening period and 14 due to other individual exclusion criteria (HIV = 1; malignoma = 2; endocarditis = 2; abusive alcohol use = 1; no surgery = 3; emergency or revisional surgery = 2; preoperative delirium = 1; dementia = 1; acute pituitary adenoma = 1) (Figure 1).

### 3.1. Legend: ECMO Extracorporeal Membrane Oxygenation

#### 3.1.1. Patients’ Characteristics

110 (37%) of 297 finally included patients were female and the median (IQR) age was 74 years (64–80) (Table 1). The majority of patients had cardiac surgery (236 patients [79.5%]) (Table 2). One-hundred-thirty patients (43.8%) had neurological complications: 129 (43.43%) POD, eight (2.7%) AIS, four (1.35%) focal or generalized seizures and 63 patients (21.21%) hallucinations (nine real-hallucinations, 52 pseudo-hallucinations and one both). All but one patient with neurological complications had a postoperative delirium except for one patient with real hallucination.

#### 3.1.2. POD Characteristics

In the 118 patients with POD but without additional seizure or AIS the diagnosis was made on day 1 in 93 patients (78.8%), in 17 patients (14.4%) on day 2 and in eight patients (6.8%) on day 3 after surgery. Forty-two of the 118 patients (35.6%) had a postoperative delirium for one day, 41 (34.7%) for two and 35 (29.7%) for three days within the screening period. The most frequent type of delirium was hyperactive delirium (*n* = 68 [57.6%]), followed by mixed (*n* = 33 [28%]) and hypoactive (*n* = 17 [14.4%]) delirium. The median severity of delirium was nine points (IQR 8–11) in CAM-S long. For 12 patients nurses reported POD only during the night. In addition to patients with delirium, 134 of the 167 patients without other neurological complications (80.2%) experienced subsyndromal delirium. For the treatment of POD 14 patients received dexmedetomidine, eight received neuroleptics (three received neuroleptics and dexmedetomidine) and seven received benzodiazepines (five of these benzodiazepines and dexmedetomidine).

#### 3.1.3. Characteristics of AIS, Seizures and Hallucinations

All AIS and seizures occurred in patients with cardiac surgery (all with procedure of cardio-pulmonary bypass (*n* = 11)). Also, all patients with AIS or seizure presented a postoperative delirium. AIS was detected at day 1 at ICU. The aetiology according to the TOAST criteria for all but one was cardio-embolism. For one patient macroangiopathy of the internal carotid artery (ICA) and cardio-embolism were competing aetiologies. A history of atrial fibrillation as competing risk factor for embolic stroke was registered in three patients. Infarction was located in the territory of the middle cerebral artery (MCA) (*n* = 5), in the brain-stem (*n* = 2) and in the cerebellum (*n* = 1). One patient showed an early onset focal seizure after bilateral infarctions in the territory of the MCA. The initial NIHSS varied between 1 and 28 points. More details are provided in Table A4.

Of the four patients with seizures, one patient had a focal motor onset seizure, one a generalized seizure and two a nonconvulsive status epilepticus. The patient with generalized seizure had a pre-diagnosed epilepsy treated with levetiracetam which was paused at the day of surgery. The other patients had no known epilepsy. All seizures occurred on the first day after surgery.

Pseudo- and real-hallucinations were detected in 63 patients (21.21%) of whom 42 (66.67%) had concomitant POD. 52 patients reported pseudo-hallucinations, nine experienced real hallucinations and one patient reported both. All but one patient with real-hallucinations showed signs of POD, whereas POD was experienced less often in patients with pseudo-hallucinations (patients without POD *n* = 20; 31.75%). Eight patients had visual and two auditive real-hallucinations. All pseudo-hallucinations were experienced visually (*n* = 53). In patients with real-hallucinations, three patients were treated with sedatives, five patients with neuroleptics and two patients were not treated with medication.

#### 3.1.4. Risk Factors for Postoperative Neurological Complications

Table 1 presents the differences between patients with or without postoperative neurological complications. The patients with postoperative neurological complications were older (*p* = 0.001), had a worse pre-interventional MoCA severity range (*p* < 0.001), more frequently chronic heart failure (*p* = 0.009), coronary heart disease (*p* = 0.008) or a history of delirium (*p* = 0.028).

In regard to neurological complications as a whole, patients with cardiac surgery (valve or bypass surgery) and percutaneous valve replacement did not differ. Nevertheless, the duration of surgery (*p* = 0.006), variance of mean arterial pressure (*p* = 0.027) and mean arterial pressure under 60 mmHg (*p* = 0.011) were associated with the occurrence of postoperative neurological complications. In addition, the number of transfusions of RBC (*p* < 0.001), platelets (*p* = 0.016) and fresh frozen plasma (*p* = 0.017) until day 3 was significantly higher compared to patients without neurological complications (Table 2).

Patients with neurological complications had in median significantly longer hospital stays (9 days, IQR 7–12 vs. 8 days IQR 7–10, *p* = 0.012). A sub-analysis of severe vs. mild postoperative delirium showed no other risk factors besides those presented. However, in multivariate analysis a higher number of RBC transfusions was an independent risk factor for severe postoperative delirium.

#### 3.1.5. Laboratory Risk Factors

**Figure 2:**
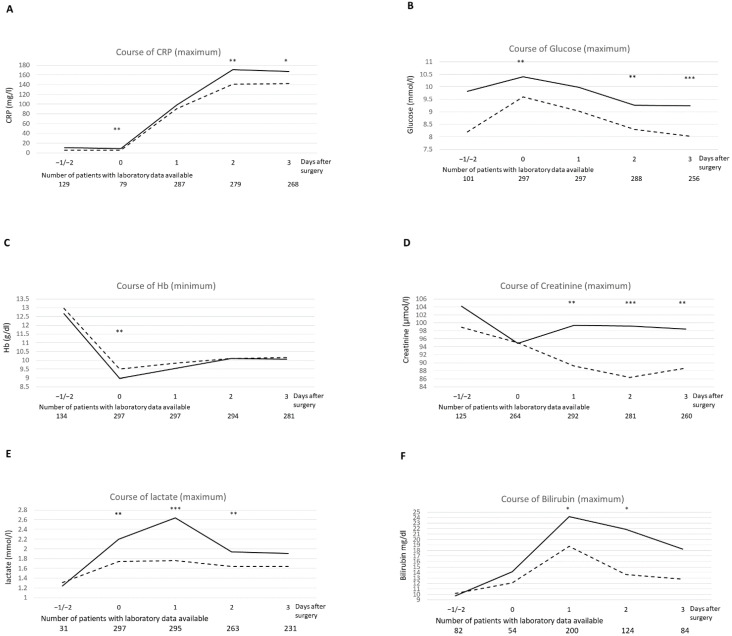
Perioperative course of biochemical markers in patients with or without neurologic complications. Legend: Temporal pattern of relevant laboratory values: CRP (**A**), Glucose (**B**), Hb (**C**), Creatinine (**D**), Lactate (**E**), Bilirubin (**F**), time points: −1 = prior to surgery, 0 = during surgery, 1 = day 1, 2 = day 2, 3 = day 3, compared using Mann-Whitney-U; *: *p* < 0.05, **: *p* < 0.01, ***: *p* < 0.001; Neurological complications: positive: _____, negative: -------.

Table A1 shows the minimum, respectively maximum of selected laboratory data for the time interval between start of surgery and postoperative day 3 for patients with and without postoperative neurological complications. Maximum CRP (*p* = 0.005), lactate (*p* < 0.001), sodium (*p* < 0.001), glucose (*p* = 0.001), creatinine (*p* = 0.003), white blood cell count (WBC) (*p* = 0.021) as well as the lowest haemoglobin (Hb) (*p* = 0.004) were significantly associated with postoperative neurological complications. Figure 2. presents the temporal pattern of relevant laboratory values for inflammation, organ dysfunctions and metabolic changes. Glucose, Hb, CRP and lactate showed significant differences between patients with and without neurological complications already at the day of surgery. The values of creatinine and bilirubin differed in the postoperative course significantly.

#### 3.1.6. Multivariable Analysis of Risk Factors for Postoperative Neurological Complications

To rule out confounding parameters, MoCA severity ranges, age, chronic heart failure, duration of surgery, number of RBC transfusions until day 3, maximum values of CRP and lactate as well as history of delirium were included in a multivariable logistic regression. The model quality (Nagelkerkes R-square) was 0.327 and a worse MoCA severity range (Exp(B) 2.042; 95% CI, 1.183–3.525, *p* = 0.010), older age (Exp(B) 1.071; 95% CI, 1.036–1.107, *p* < 0.001), the number of RBC transfusions until postoperative day 3 (Exp(B) 1.157; 95% CI, 1.030–1.300, *p* = 0.014), history of heart failure (Exp(B) 1.985; 95% CI, 1.130–3.487, *p* = 0.017) and increased CRP levels (Exp(B) 1.004; 95% CI, 1.000–1.008, *p* = 0.037) remained independently associated with the occurrence of neurological complications.

Focussing on preoperative risk factors for the occurrence of neurological complications, a preoperative-model was accomplished. MoCA severity range, age, history of heart failure, history of delirium and type of surgery were tested. The model quality (Nagelkerkes R-square) was 0.212 and a worse MoCA severity range (Exp(B) 2.218; 95% CI, 1.324–3.716, *p* = 0.002), older age (Exp(B) 1.061; 95% CI, 1.029–1.093, *p* < 0.001), history of heart failure (Exp(B) 2.128; 95% CI, 1.261–3.593, *p* = 0.005) and type of surgery (Exp(B) 4.865; 95% CI, 2.338–10.125, *p* < 0.001) were associated with the occurrence of neurological complications.

#### 3.1.7. Hallucinations as Postoperative Neurological Complications

A univariate analysis of patients with or without postoperative hallucinations showed preoperative arterial hypertension (*p* < 0.001), history of neurotrauma (*p* < 0.001), preoperative antidepressants (*p* = 0.036), intraoperatively increased sedatives (*p* = 0.029) and a lower intraoperative haemoglobin value (*p* = 0.019) as risk factors. To exclude confounding parameters, arterial hypertension, history of neurotrauma, preoperative antidepressants, intraoperatively sedatives as well as the lowest intraoperative haemoglobin value were included in a multivariable logistic regression. The model quality (Nagelkerkes R-square) was 0.219 and arterial hypertension (Exp(B) 5.338; 95% CI, 2.767–10.299, *p* < 0.001) as well as history of neurotrauma (Exp(B) 3.391; 95% CI, 1.811–6.349, *p* < 0.001) remained independently associated with the occurrence of neurological complications.

#### 3.1.8. Comparison of Percutaneous Valve Replacement and Cardiac Surgery

36.1% (*n* = 22) of the 61 patients with percutaneous valve replacement and 45.8% (*n* = 108) of the 236 patients with cardiac surgery developed neurological complications (*p* = 0.174). The patients with percutaneous valve replacement were significantly older (*p* < 0.001), suffered more often from atrial fibrillation (*p* = 0.017) or heart failure (*p* = 0.001), had a higher baseline MoCA severity range (*p* = 0.001) and higher American Society of Anaesthesiologists (ASA)-classification (*p* < 0.001). Patients with cardiac surgery were more frequently male (*p* < 0.001) and more frequently had a history of myocardial infarction (MI) (*p* = 0.010). Also, the duration of surgery was longer (*p* < 0.001), variance of MAP (*p* < 0.001) higher, the number of RBC transfusions (*p* < 0.001) higher, a MAP under 60 mmHG (*p* < 0.001) and longer stay in hospital longer (*p* < 0.001) occurred more often. The maximum of CRP (*p* < 0.001), lactate (*p* < 0.001), potassium (*p* < 0.001) and calcium (*p* < 0.001) were significantly higher in patients with cardiac surgery. See Appendix A for more details (Table A2).

## 4. Discussion

In this study, we prospectively evaluated a large cohort of 297 patients undergoing percutaneous valve replacement or cardiac surgery for prevalence of postoperative neurological complications. The neurological events and their course were observed in detail. In addition, the corresponding risk factors were analysed. The rate of neurological complications in our cohort was high in both, percutaneous valve replacement as well as cardiac surgery. POD was the most frequent neurological complication with 43.43% (*n* = 129), and all patients with AIS, seizure or real hallucinations but one also had POD. The frequency of POD, hallucinations and AIS was in the same range as presented in previous studies of patients with cardiac surgery [2,3]. In our cohort, impaired cognitive function, a higher age, increased postoperative inflammation as measured by biochemical blood markers as well as a higher number of RBC transfusions were independently associated with an increased risk of postoperative neurological complications. This is in accordance to the metaanalysis of Bramley et al. with trauma or orthopaedic, cardiac and vascular surgery which showed that increasing age, pre-existing cognitive impairment, cerebrovascular disease and intraoperative blood transfusion are substantial risk factors for POD representing by far the most frequent neurological complications [15].

Our finding of an independent association of increased inflammation levels (CRP values) after surgery with neurological complications, supports the inflammation explanatory approach of the pathogenesis of POD. It is hypothesized that surgery and anaesthesia trigger leakiness of the blood-brain barrier (BBB) allowing perioperatively increased inflammation mediators to cross this barrier [16]. Hovens et al. showed in rats an increased level of neuroinflammation from immediately after cardiac surgery until up to two weeks later [17]. The study also demonstrated the influence of increased neuroinflammation on postoperative cognitive performance. All rats had an impaired spatial memory and were impaired in spatial learning and object recognition after cardiac surgery [17]. Also, the significant association of RBC transfusions and occurrence of neurological complications encourages the inflammation hypothesis. Transfusions are well known as trigger for systemic inflammation [18]. However, transfusions are linked to several factors such as intraoperative blood loss and haemodynamic instability which could confound the association. Also, temporary hypoxemia in case of severe blood loss enhances inflammatory responses and according BBB leakages. In addition, CRP and lactate were significantly higher in patients with neurological complications postoperatively. In comparison of patients with cardiac surgery or percutaneous valve replacement, CRP and lactate were higher in the former group, which could demonstrate the influence of the more invasive cardiac surgery. The significant differences of laboratory values like creatinine for renal failure and bilirubin for liver failure indicate differences in the grade of organ dysfunctions which increase the risk of POD. Taken together our findings support the inflammation and organ dysfunction hypothesis as an important pathogenic mechanism of POD. Minimal-invasive surgery could be a possibility to reduce postoperative inflammation [19]. But further studies which analyse preventive risk-strategies and the prevalence of AIS, seizures and hallucinations after surgery in a larger number of patients are needed.

When comparing patients with cardiac surgery to those with percutaneous valve replacement in regard to postoperative neurological complications, the latter showed a considerably lower occurrence of neurological complications (46.7% vs. 36%). However, it should be considered that the number of patients with percutaneous valve replacement was relatively small. Patients with percutaneous valve replacement had a severe preoperative risk profile, like higher age (83 years vs. 70.5 years) and preoperative cognitive impairment, both known risk factors for POD. However, other significant perioperative factors like postoperative inflammation or transfusions connected with more invasive surgery seem to have a greater influence for developing postoperative neurological complications and should receive focus in further prospective studies. Since risk factors divide in modifiable (e.g., transfusions) and non-modifiable (such as age or preoperative cognitive impairment), each patient should be evaluated individually in regard to pre-existing conditions and intended type of planned surgery. In previous studies, the incidence of postoperative delirium after percutaneous valve replacement was between 0% to 44.6% [20]. In comparison, our study shows a relatively high number of these patients (36.1%). However, our incidence of postoperative delirium is higher in patients undergoing cardiac surgery, which is consistent with previous prospective studies [21] while the difference between the two groups of patients with postoperative delirium is smaller in our study (42.7% vs. 36.1% to 51% vs. 15%) [21]. When the risk profile of patients is evaluated carefully, there may be a chance to prevent or at least reduce the extent of POD and its consecutive complications. However, most of the risk factors are non-modifiable. Hence, it is important to detect postoperative neurological complications early with a structured assessment for better treatment options.

Regarding modifiable risk factors for the occurrence of delirium and postoperative AIS in patients with cardiac surgery, previous meta-analysis has shown that a restrictive transfusion regimen is at least equivalent to a liberal transfusion management [22]. Patel et al. even found an increased 30day-mortality for a liberal transfusion management [23]. Our result of the independent association of RBC transfusion with an increased risk of postoperative neurological complications may indicate the usefulness of a restrictive transfusion management but we cannot support this thesis because transfusion was no primary outcome in our study. Also, the need for transfusion simultaneously indicates severe courses with potentially higher levels of inflammation and comparatively increased hypoxemia. We also analysed the influence of the intraoperative MAP on postoperative neurological complications with different established perspectives by using the mean MAP, MAP under 60mmHg and variance of MAP [24]. None of these parameters was identified as an independent risk factor for the development of postoperative neurologic complications.

Hallucinations represented the second most frequent postoperative neurological complication in our cohort. The incidence of 21.21% was similar to previous studies [2,25] and we could confirm this in a larger prospective cohort. Visual hallucinations presented the largest portion in accordance with earlier studies for patients after cardiac surgery [2] and non-psychiatric patients with delirium [25]. Real hallucinations as well as pseudo-hallucinations have relevant clinical implications: patients with hallucinations are at a higher risk for a difficult postoperative recovery like post intensive care syndrome (PICS) [26], which is associated with longer ICU-stay and postoperative complications [27]. Moreover, the patients with hallucinations are more likely to harm themselves [28]. In our cohort, arterial hypertension and history of neurotrauma were detected as new risk factors for postoperative hallucinations. The retrospective study from Tachibana et al. showed that use of alcohol, benzodiazepine withdrawal, use of angiotensin II receptor blocker and dopamine receptor agonists are more frequent in patients with postoperative hallucinations [25], whereas Ottens et al. did not find any preoperative risk factors [2]. Of note, in our study patients with alcohol or benzodiazepine abuse were excluded.

Four patients had a seizure or status epilepticus at the first postoperative day after cardiac surgery in our study. It is quite conceivable that the surgery has an effect as metabolic or inflammatory trigger for seizures. One of the patients had pre-diagnosed epilepsy and the pharmacotherapy was paused for the day of surgery. That shows the importance of continuous application of the anticonvulsant medication also at the day of surgery. Another patient had the seizure after AIS in the territory of MCA, explaining an acute brain lesion as trigger for the seizure. But we cannot exclude that silent brain infarction was the trigger for the other seizures, because imaging was not conducted. Palanca et al. showed that non-convulsive status epilepticus could be an underestimated aetiology for patients with postoperative delirium after cardiac surgery [29]. Not to forget, the delirium could manifest during the ictal period or postictally and non-convulsive seizures could present as delirium, respectively. Therefore, a postoperative EEG diagnostic for patients with symptoms of delirium should be considered if in doubt.

The predominant cardio-embolic aetiology in patients with AIS after cardiac surgery shows that the direct manipulation at heart and major thoracic vessels as well as the cardio-pulmonary bypass are important sources of AIS. Although we did not observe AIS in patients with percutaneous valve replacement, previous studies showed that a silent cerebral injury occurs frequently [30]. Since the postoperative neurological imaging (magnetic resonance imaging) is restricted due to the temporary pacemaker, the thoughtful examination for early detection and treatment is necessary. In our study, all patients with postoperative AIS fulfilled simultaneously the criteria for a postoperative delirium. Shaw et al. demonstrated that deliria are frequently associated with AIS without the influence of surgeries [31]. Therefore, in our study the detected postoperative delirium in patients with AIS could be triggered by the AIS themselves. Hence, patients with POD, change or reduction of consciousness should always be examined for acute focal neurological deficits, however this is more difficult in non-compliant patients.

Our structured assessment detected a high rate of postoperative neurological complications which have been shown in former studies to result in poor outcome and higher in-hospital mortality [3,8]. Patients with percutaneous valve replacement had a similar frequency of POD but no AIS and seizures in comparison to patients with cardiac surgery, although the patients with percutaneous valve replacement had a higher preoperative risk profile. Accordingly, turning to minimally invasive surgical and interventional techniques may be an appropriate strategy to reduce neurological complications.

## 5. Limitations

Though this is one of a few prospective studies upon neurological complications after cardiac surgery and percutaneous valve replacement the relatively small number of patients with percutaneous valve replacement can be considered as limitation. We probably did not discover all POD episodes as patients can develop delirium after the third postoperative day [32]. Furthermore, the incidence of hallucinations might be underrated because patients with a severe POD were impaired in their ability to communicate and patients with real hallucinations could neither recognise nor remember them.

## 6. Conclusions

POD is the most frequent postoperative neurological complication in patients with cardiac surgery or percutaneous valve replacement. AIS, focal or generalized seizures and hallucinations are also of importance, but occur less frequently. Patients with percutaneous valve replacement had no AIS or seizures. The results suggest that patients with neurological complications have a preoperative risk profile including older age, history of heart failure and cognitive impairment. But a complicated intra- and postoperative course with intense inflammatory response and diverse organ dysfunction might be a more important trigger for developing postoperative neurological complications.

## Figures and Tables

**Table 1:** Clinical characteristics of patients with cardiac surgery or percutaneous valve replacement with or without neurological complications.

	Total Cohort (*n* = 297 pat.)	Neurological Complications (*n* = 130 pat.)	No Neurological Complications (*n* = 167 pat.)	*p*-Value
Sex				0.446
female	110 (37%)	45(34.6%)	65 (38.9%)	
male	187 (63%)	85 (65.4%)	102 (61.1%)	
Age [years], median (IQR)	74 (64–80)	76 (69.75–80.25)	71 (62–79)	**0.001 ^+^**
BMI [kg/m²], mean (SD)	27.48 (5.23)	27.182 (4.25)	27.71 (5.76)	0.643 ^+^
DM	79 (26.6%)	44 (33.8%)	35 (21%)	**0.017**
CHD	262 (88.6%)	122 (93.8%)	140 (83.8%)	**0.008**
HF	170 (57.2%)	86 (66.2%)	84 (50.3%)	**0.006**
aHTN	68 (22.9%)	34 (26.2%)	34 (20.4%)	0.238
Obesity	74 (24.92%)	32 (24.6%)	42 (25.1%)	0.916
History of MI	46 (15.5)	26 (20%)	20 (12%)	0.058
History of AF	85 (28.62%)	38 (29.2%)	47 (28.1%)	0.837
History of chronic alcoholism	26 (8.75%)	11 (8.5%)	15 (9%)	0.875
History of neurotrauma	105 (35.35%)	43 (33.1%)	62 (37.1%)	0.469
History of AIS	32 (10.8%)	18 (13.8%)	14 (8.4%)	0.132
History of delirium	21 (7.1%)	14 (10.8%)	7 (4.2%)	**0.028**
History of surgery < 6 months	20 (6.73%)	11 (8.5%)	9 (5.4%)	0.295
History of depression	21 (7.07%)	9 (6.9%)	12 (7.2%)	0.930
History of malignoma > 2 years	35 (11.78%)	19 (14.6%)	16 (9.6%)	0.182
MoCA severity range preoperative, median (IQR)	2 (1–2)	2 (2–2)	2 (1–2)	<0.001 *
PHQ-2, median (IQR)	0 (0–1)	0 (0–1)	0 (0–1)	0.121 *
MST, median (IQR)	0 (0–1)	0 (0–1)	0 (0–1)	0.365 *
ASA-classification, median (IQR)	3 (3–3)	3 (3-3)	3 (3–3)	0.472 *
Pre-medication sedatives or benzodiazepines	10 (3.37%)	5 (3.8%)	5 (3%)	0.753
Stay in hospital in days, median (IQR)	8 (7–11)	9 (7–12)	8 (7–10)	**0.012 ^+^**

Data are presented as mean ± standard deviation, median (interquartile range), or proportions, and compared using chi-square tests, Mann-Whitney-U (^+^) and Fisher’s exact test (*), respectively. Statistically significant results are shown in bold, *p* < 0.05 was considered statistically significant. DM Diabetes Mellitus, BMI Body-Mass-Index, CHD coronary heart disease, HF heart failure, aHTN arterial hypertonia, MI myocardial infarction, AF atrial fibrillation, AIS acute ischemic stroke, MoCA Montreal Cognitive Assessment severity ranges: 1 = no cognitive impairment (26–30 points), 2 = mild cognitive impairment (18–25 points), 3 = moderate cognitive impairment (10–17 points) and 4 = severe cognitive impairment (<10 points), PHQ-2 Patient Health Questionnaire-2, MST Malnutrition Screening Tool, ASA American Society of Anaesthesologists.

**Table 2:** Perioperative parameters for patients with or without neurological complications.

	Total Cohort (*n* = 297 pat.)	Neurological Complications (*n* = 130 pat.)	No Neurological Complications (*n* = 167 pat.)	*p*-Value
Percutaneous valve replacement	61 (20.5%)	22 (16.9%)	39 (23.4%)	0.174
Cardiac surgery	236 (79.5%)	108 (83.1%)	128 (76.6%)	
Premedication midazolam	77 (25.9%)	30 (23.1%)	47 (28.1%)	0.323
Premedication midazolam standardized, 7.5 mg 3.5 mg	35 (11.8%) 44 (14.8%)	12 (9.2%) 20 (15.4%)	23 (13.8%) 24 (14.4%)	0.483
Duration of surgery [minutes], median (IQR)	189 (135.5–239)	199 (157.75–248.5)	182 (111–223)	**0.006 ^+^**
Cardio-pulmonary-bypass time [minutes], mean (SD) ^1^	116.62 (47.985)	123.69 (51.55)	110.8 (44.23)	**0.035 ^+^**
Aorta clamping time, mean (SD) ^2^	68.92 (31.68)	71.84 (32.55)	66.46 (30.86)	0.186 ^+^
MAP under 60 mmHg	260 (87.5%)	121 (93.1%)	139 (83.2%)	**0.011**
MAP minimum [mmHg], mean (SD)	34.75 (13.42)	32.32 (11.95)	36.64 (14.21)	**0.016 ^+^**
MAP mean, mean (SD)	71.84 (11.21)	71.10 (9.53)	72.44 (12.36)	0.408 ^+^
MAP variance [mmHg], mean (SD)	190.90 (93.42)	203.86 (99.35)	180.82 (87.50)	**0.027 ^+^**
Administration of glucocorticoids	11 (3.7%)	3 (2.3%)	8 (4.8%)	0.358 *
Medication of vasopressor	179 (60.3%)	81 (62.3%)	98 (58.7%)	0.526
Medication of inotropica	58 (19.5%)	31 (23.8%)	27 (16.2%)	0.098
Number of RBC transfusion (intraop.), median (IQR)	0 (0–2)	0 (0–2)	0 (0–0)	**<0.001** ^+^
Administration of FFP (intraop.)	14 (4.7%)	9 (6.9%)	5 (3%)	0.113
Administration of thrombocyte concentrate (intraoperative)	30 (10.1%)	18 (13.8%)	12 (7.2%)	0.059
Revisional surgery	15 (5.1%)	8 (6.2%)	7 (4.2%)	0.444
Number of RBC transfusions (total), median (IQR)	2 (0–4)	3 (0.75–6)	0 (0–3)	**0.028 ^+^**
Administration of FFP (total)	45 (15.2%)	27 (20.8%)	18 (10.8%)	**0.017**
Administration of thrombocyte concentrate (total)	68 (22.9%)	38 (29.2%)	30 (18%)	**0.022 ^+^**
Neuroleptics postoperative	15 (5.05%)	13 (10%)	2 (1.2%)	**0.001**
Opioids postoperative	250 (84.17%)	114 (87.7%)	136 (81.4%)	0.143
Sedatives postoperative	228 (76.8%)	107 (82.3%)	121 (72.5%)	**0.046**
AF postoperative	64 (21.55%)	32 (24.6%)	32 (19.2%)	0.257

^1^ only patients with cardiac surgery and use of cardio-pulmonary-bypass (*n* = 206); ^2^ only patients with cardiac surgery and use of aorta clamping (*n* = 202). Data are presented as mean ± standard deviation, median (interquartile range), or proportions, and compared using chi-square tests, ^+^ Mann-Whitney-U and * Fisher’s exact test, respectively. Statistically significant results are shown in bold, *p* < 0.05 was considered significant. MAP mean arterial pressure, RBC red blood cell, FFP fresh frozen plasma, AF atrial fibrillation.

## Data Availability

The data that support the findings of this study are available from the corresponding author upon reasonable request.

## Abbreviations

ACI	arteria carotid interna
AIS	acute ischemic stroke
ASA	American Society of Anaesthesiologists
CAM	Confusion Assessment Method
CAM-ICU	Confusion Assessment Method for intensive care unit
ECMO	Extracorporeal membrane oxygenation
EEG	Electroencephalography
IQR	Interquartile range
MI	Myocardial infarction
MoCA	Montreal Cognitive Assessment
MRI	Magnetic resonance imaging
MST	Malnutrition Screening Tool
NIHSS	National Institutes of Health Stroke Scale
NRS	Numeric pain rating scale
PHQ-2	Patient Health Questionnaire-2
POD	postoperative delirium
RBC	Red blood cell
SD	Standard deviation

## Appendix A

**Table A1:** Postoperative biochemical markers in patients with or without neurological complications.

	Total Cohort (*n* = 297 pat.)	Neurological Complications (*n* = 130 pat.)	No Neurological Complications (*n* = 167 pat.)	*p*-Value
CRP max. [mg/L], mean (SD)	164.25 (90.3)	180.87 (87.9)	151.31 (90.27)	**0.005 ^+^**
Lactate max. [mmol/L], mean (SD)	2.74 (1.76)	3.24 (2.16)	2.35 (1.24)	**<0.001 ^+^**
Hb min. [g/dl], mean (SD)	8.56 (1.67)	8.33 (1.73)	8.75 (1.6)	**0.004 ^+^**
Sodium max. [mmol/L], mean (SD)	143.44 (3.15)	144.3 (3.85)	142.77 (2.27)	**<0.001 ^+^**
Sodium min. [mmol/L], mean (SD)	134.9 (2.95)	134.89 (3.41)	134.91 (2.54)	0.316 ^+^
Potassium max. [mmol/L], mean (SD)	5.57 (0.81)	5.7 (0.85)	5.48 (0.76)	0.141 ^+^
Potassium min. [mmol/L], mean (SD)	3.71 (0.35)	3.72 (0.38)	3.7 (0.32)	0.397 ^+^
Glucose max. [mmol/L], mean (SD)	11.11 (2.95)	11.7 (3.1)	10.66 (2.76)	**0.001 ^+^**
Glucose min. [mmol/L], mean (SD)	5.2 (1.04)	5.26 (1.04)	5.16 (1.04)	0.846 ^+^
Creatinine max. [μmol/L], mean (SD)	104.71 (61.5)	109.2 (38.96)	101.22 (74.45)	**0.003** ^+^
Leucocytes max. [cells/µL], mean (SD)	12.97 (4.52)	13.68 (4.78)	12.41 (4.23)	**0.021** ^+^
Calcium max. [mmol/L], mean (SD)	1.9 (0.43)	1.91 (0.40)	1.91 (0.45)	0.474 ^+^
Calcium min. [mmol/L], mean (SD)	1.13 (0.07)	1.12 (0.08)	1.13 (0.07)	0.743 ^+^

Data are presented as mean ± standard deviation and compared. using ^+^ Mann-Whitney-U; Statistically significant results are shown in bold, *p* < 0.05 was considered significant. Max. maximum, min. minimum, Hb Haemoglobin, CRP C-reactive protein, WBC white blood cells, Ca Calcium.

**Table A2:** Clinical characteristics in patients with percutaneous valve replacement and cardiac surgery.

	Percutaneous Valve Replacement (*n* = 61)	Cardiac Surgery (*n* = 236)	*p*-Value
Neurological complications, postoperative	22 (36.1%)	108 (45.8%)	0.174
Hallucinations, postoperative	10 (16.4%)	53 (22.5%)	0.302
Isolated POD, postoperative	22 (36.1%)	96 (42.7%)	0.353
AIS, postoperative	0 (0%)	8 (3.4%)	0.368 *
Focal/generalized seizure, postoperative	0 (0%)	4 (1.7%)	0.585 *
Sex, female	36 (59%)	74 (31.4%)	**<0.001 ^+^**
Age [years], median (IQR)	83 (79–87)	70.5 (63–77)	**<0.001**
aHTN, preoperative	18 (29.5%)	50 (21.2%)	0.168
DM	17 (27.9%)	62 (26.3%)	0.801
History of MI	3 (4.9%)	43 (18.2%)	**0.010**
History of AF	25 (41%)	60 (25.4%)	**0.017**
Coronary heart disease	54 (88.5%)	208 (88.1%)	0.933
HF	46 (75.4%)	124 (52.5%)	**0.001**
History of POD	5 (8.2%)	16 (6.8%)	0.700
MoCA severity range preoperative, Median (IQR)	2 (2–2)	2 (1–2)	**0.001 ***
History of neurotrauma	19 (31.1%)	86 (36.4%)	0.441
PHQ-score, median (IQR)	0 (0–1)	0 (0–1)	0.890 *
Malnutrition-score, median (IQR)	0 (0–1)	0 (0–1)	0.451 *
Premedication sedatives	5 (8.2%)	72 (30.5%)	**<0.001**
ASA-classification, median (IQR)	3 (3–4)	3 (3–3)	**<0.001 ***
Duration of surgery [minutes], median (IQR)	55 (43.5–76.5)	205 (172–249)	**<0.001 ^+^**
MAP intraoperative min. [mmHg], mean (SD)	51.97 (13.52)	30.3 (9.13)	**<0.001 ^+^**
MAP mean [mmHg], mean (SD)	85.47 (16.93)	68.32 (4.96)	**<0.001 ^+^**
MAP intraoperative variance [mmHg], mean (SD)	14.64 (98.21)	203.12 (88.32)	**<0.001 ^+^**
MAP under 60mmHg	28 (45.9%)	232 (98.3%)	**<0.001**
Number of RBC transfusions (intraop.), median (IQR)	0 (0–0)	0 (0–2)	**<0.001 ^+^**
Administration of FFP (intraop.)	0 (0%)	14 (5.9%)	0.082 *
Administration of thrombocyte concentrates (intraop.)	0 (0%)	30 (12.7%)	**0.003**
AF postoperative	7 (11.5%)	57 (24.2%)	**0.032**
Number of RBC transfusions (total),median (IQR)	0 (0–0)	2 (0–4)	**<0.001 ^+^**
Administration of FFP (total)	0 (0%)	45 (19.1%)	**<0.001**
Administration of thrombocyte concentrates (total)	0 (0%)	68 (28.8%)	**<0.001**
Stay in hospital in days, median (IQR)	7 (6–7)	9 (7.5–12)	**<0.001 ^+^**
CRP max. [mg/L], mean (SD)	47.95 (34.94)	194.31 (74.41)	**<0.001 ^+^**
Lactate max. [mmol/L], mean (SD)	1.78 (0.71)	2.98 (1.86)	**<0.001 ^+^**
Hb min. [g/dl], mean (SD)	9.63 (1.83)	8.3 (1.5)	**<0.001 ^+^**
Sodium max. [mmol/L], mean (SD)	141.92 (2.74)	143.83 (3.13)	**<0.001 ^+^**
Glucose max. [mmol/L], mean (SD)	9.91 (2.3)	11.42 (3.02)	**<0.001 ^+^**
Glucose min. [mmol/L], mean (SD)	5.71 (0.93)	5.07 (1.02)	**<0.001 ^+^**
Potassium max. [mmol/L], mean (SD)	4.64 (0.37)	5.82 (0.70)	**<0.001 ^+^**
Calcium max. [mmol/L], mean (SD)	1.3 (0.17)	2.1 (0.32)	**<0.001 ^+^**
Creatinine max. [μmol/L], mean (SD)	104.72 (33.06)	104.70 (66.92)	0.238 ^+^

Data are presented as mean ± standard deviation, median (interquartile range), or proportions, and compared using chi-square test, ^+^ Mann-Whitney-U and * Fisher’s exact test, respectively. Statistically significant results are shown in bold, *p* < 0.05 was considered significant. Max. maximum, min. minimum, Intraop. Intraoperative, POD postoperative Delirium, DM Diabetes Mellitus, BMI Body-Mass-Index, CHD chronic heart disease, HF heart failure, aHTN arterial hypertonia, MI myocardial infarction, AF atrial fibrillation, AIS acute ischemic stroke, MoCA Montreal Cognitive Assessment severity ranges: 1 = no cognitive impairment (26–30 points), 2 = mild cognitive impairment (18–25 points), 3 = moderate cognitive impairment (10–17 points), PHQ-2 Patient Health Questionnaire-2, MST Malnutrition Screening Tool, ASA American Society of Anaesthesologists, MAP mean arterial pressure, RBC red blood cell, FFP fresh frozen plasma.

**Table A3:** Clinical characteristics and risk factors in patients with and without hallucinations.

	Hallucinations Positive (*n* = 63 pat.)	Hallucinations Negative (*n* = 234 pat.)	*p*-Value
Sex, female	18 (28.6%)	92 (39.3%)	0.117
Age [years], median (IQR)	72 (65–79)	74 (64–80)	0.765
BMI [kg/m²], mean (SD)	26.96 (5.01)	27.62 (5.3)	0.404
aHTN preoperative	29 (46%)	39 (16.7%)	**<0.001**
History of neurotrauma	34 (54%)	71 (30.3%)	**<0.001**
History of alcohol abuse	7 (11.1%)	19 (8.1%)	0.456
Benzodiazepines, preoperative	1 (1.6%)	2 (0.9%)	1.000
Antidepressants, preoperative	6 (9.5%)	7 (3%)	**0.036** *
MoCA severity range preoperative, Median (IQR)	2 (1–2)	2 (1–2)	0.744 *
sedatives intraoperative	58 (92.1%)	188 (80.3%)	**0.029**
Hb min. [g/dL], mean (SD), intraoperative	8.86 (1.4)	9.35 (1.7)	**0.019 ^+^**
sodium max. at day 1 postoperative [mmol/L], mean (SD)	142.76 (3.8)	141.74 (3.38)	**0.044 ^+^**

Data are presented as mean ± standard deviation, median (interquartile range), or proportions, and compared using chi-square tests, ^+^ Mann-Whitney-U and * Fisher’s exact test, respectively. *p* < 0.05 was considered statistically significant. Statistically significant results are shown in bold. max. maximum, min. minimum, BMI Body-Mass-Index, aHTN arterial hypertonia, MoCA Montreal Cognitive Assessment severity ranges: 1 = no cognitive impairment (26–30 points), 2 = mild cognitive impairment (18–25 points), Hb Haemoglobi.

**Table A4:** Patients with postoperative acute ischemic stroke: aetiology, severity and risk factors.

Patient	Type of Surgery	Age	Sex	Postoperative Day of Detection	Localisation Infarction	TOAST Criteria (Aetiology)	Stenosis of Cerebral Vessels	NIHSS Baseline	mRS Baseline	mRS at Discharge	Cerebrovascular Risk Factors	Antithrombotic Treatment
1	Valve surgery with cardio-pulmonary-bypass	74	m	Day 1	Brainstem	Cardio-embolic	-	10	5	4	DM, aHTN, AF, CHD, HLP	Phenprocoumon
2	Valve surgery with cardio-pulmonary-bypass	74	f	Day 1	Territory of MCA (left)	Cardio-embolic	ACI (bifurcation) right and Left (low-grade),ACI (siphon, moderate)	1	2	2	AF, CHD, HF	Phenprocoumon
3	Valve and bypass surgery with cardio-pulmonary-bypass	78	m	Day 1	Territory of MCA (double-sided)	Cardio-embolic	ICA, right (high-grade)	29	5	5	AIS, CHD, HF, pAOD, MI, HF	Phenprocoumon
4	Valve surgery with cardio-pulmonary-bypass	78	m	Day 1	Territory of MCA (right)	Cardio-embolic	-	3	3	1	ACI, aHTN, AF, CHD, HF, HLP, adiposis, sclerosis	Phenprocoumon
5	Valve and bypass surgery with cardio-pulmonary-bypass	74	m	Day 1	Brain stem	Makro-angiopathy competing with cardio-embolic	VA left (high-grade), BA (low-grade), ACI right (moderate)	12	5	3	AIS, DM, aHTN, CHD, HF, pAOD, HLP, history of carotis TEA (right)	Phenprocoumon
6	Valve surgery with cardio-pulmonary-bypass	67	m	Day 1	Territory of MCA (left)	Cardio-embolic	-	5	4	1	aHTN, AIS, CHD, HF, HLP	Phenprocoumon
7	Bypass surgery with cardio-pulmonary-bypass	77	m	Day 1	Territory of MCA (left)	Cardio-embolic	-	4	3	0	aHTN, CHD, HF, HLP	ASA
8	Bypass surgery with cardio-pulmonary-bypass	78	m	Day 1	Cerebellum (double-sided) brain stem	Cardio-embolic	ACI right (high-grade) and left (moderate)	28	5	5	aHTN, AIS, CHD, pAOD, HF, MI, smoking cigarettes	ASA

No CTA because of chronic renal failure. ACI arteria carotid interna. aHTN arterial hyertonia. AIS ischemic stroke. ASA acetylsalicylic acid. BA basilar artery. Carotis TEA Carotis thromboendarterectomy. CHD chronic heart disease. DM Diabetes Mellitus. HF heart failure. HLP hyperlipoproteinemia. MI myocardial infarction. pAOD peripheral arterial occlusive disease. VA vertebral artery.
